# Prevalence and Self-Perceived Experiences With the Use of Hormonal Contraceptives Among Competitive Female Cross-Country Skiers and Biathletes in Norway: The FENDURA Project

**DOI:** 10.3389/fspor.2022.873222

**Published:** 2022-04-14

**Authors:** Tina P. Engseth, Erik P. Andersson, Guro S. Solli, Bente Morseth, Tor Oskar Thomassen, Dionne A. Noordhof, Øyvind Sandbakk, Boye Welde

**Affiliations:** ^1^School of Sport Sciences, UiT, The Arctic University of Norway, Tromsø, Norway; ^2^Department of Sports Science and Physical Education, Nord University, Bodø, Norway; ^3^Department of Neuromedicine and Movement Science, Centre for Elite Sports Research, Norwegian University of Science and Technology, Trondheim, Norway

**Keywords:** combined hormonal contraceptives, endurance, female athletes, hormonal contraceptives, progestin-only hormonal contraceptives

## Abstract

**Purpose:**

To investigate the prevalence of hormonal contraceptive (HC) use by female cross-country (XC) skiers and biathletes competing at a national and/or international level, their reasons for HC use, and to compare negative symptoms related to the HC-/menstrual cycle in HC users and non-HC users. Additionally, to characterize the self-perceived influence of HC use on training and performance.

**Methods:**

A total of 113 Norwegian competitive XC skiers and biathletes completed an online questionnaire including both closed and open-ended questions. The questions were designed to assess the type of HC, reasons for use, self-reported negative symptoms related to HC-/menstrual cycle, as well as athletes' experiences regarding how HC use affects training and performance.

**Results:**

In total, 68% of all the athletes used HC, with 64 and 36% of them using a progestin-only and combined type HC, respectively. Non-contraceptive reasons for HC use were reported by 51% of the progestin-only HC users vs. 75% of the combined HC users (*P* = 0.039), with reduction of negative menstrual-related symptoms as the most common reason. Of the athletes reporting regular withdrawal bleedings in connection to HC use, 80% of the progestin-only and 86% of combined HC users experienced negative menstrual-related symptoms, which was comparable to the non-HC group (86%). The majority (81%) of HC users experienced solely positive, or no effect, of HC use on training and performance, with no differences between progestin-only and combined HC users (*P* = 0.942).

**Conclusions:**

In total, 68% of the XC skiers and biathletes used HC, with the highest proportion (64%) using a progestin-only HC. Many athletes used HC to manipulate their menstrual cycle due to perceived negative menstrual-related symptoms that interfered with their training sessions and/or competitions.

## Introduction

Hormonal contraceptives (HCs) are exogenous hormones used to alter endogenous sex hormone concentrations to prevent pregnancy or for medical and/or health-related purposes (Davis and Westhoff, 2001; Bitzer and Simon, 2011; Burke, 2011; Shulman, 2011). HCs can be classified into two main types, progestin-only or combined, based on their concentration of synthetic estrogen and progestin (Burke, 2011; Shulman, 2011). Progestin-only HCs include oral contraceptives (OCs; mini pills), implants, injections, and intrauterine systems (IUSs) (Burke, 2011), while combined HCs contain both synthetic estrogen and progestin and include OCs, transdermal patches, and vaginal rings as delivery methods (Shulman, 2011).

Approximately 40% of women (15–49 years) in the general Nordic population have been reported to use HCs, with the most common delivery method being combined OCs (Lindh et al., 2017). However, recent data from the Norwegian Prescription Database shows a rapid increase in the use of long-acting progestin-only HCs (i.e., implants and IUS) during the past 3 years (Furu et al., 2021). In the athlete population, approximately half of the respondents from various sports in the United Kingdom, Ireland, Denmark, and Norway reported hormonal contraceptive (HC) use (Martin et al., 2018; Oxfeldt et al., 2020; Solli et al., 2020; Nolan et al., 2022), with athletes in technical sports (e.g., golf, table tennis etc.) showing a higher proportion of HC use (80%) compared to athletes competing in endurance sports (50%) (Oxfeldt et al., 2020). Overall, combined HCs were more commonly used than progestin-only HCs (Martin et al., 2018; Oxfeldt et al., 2020; Solli et al., 2020). Although these studies were recently published, it is unclear if the rapid shift toward the use of long-acting progestin-only HCs, as reported by Furu et al. (2021), is also present in the athletic population.

Although HCs are mainly used to prevent pregnancy, both types of HCs are also used for other medical or health-related purposes, such as reducing premenstrual syndrome, premenstrual dysphoric disorder, anemia, mild-to-moderate acne, and other negative menstrual-related symptoms (Burke, 2011; Shulman, 2011). In addition, hormonal contraceptives are widely used to manipulate menstruation in recreationally active and competitive women (Schaumberg et al., 2018). In athletes, the consensus from previous research is that prediction and manipulation of menstruation are the most common non-contraceptive reasons, and positive side-effects, for using HCs (Martin et al., 2018; Armour et al., 2020; Elliott-Sale et al., 2020; Oxfeldt et al., 2020; Clarke et al., 2021), even though a meta-analysis showed that OC use results in a slightly lower performance compared to a natural menstrual cycle (Elliott-Sale et al., 2020). When Armour et al. (2020) investigated why Australian team-sports athletes chose to manipulate their menstruation, “convenience” and “reducing the impact on sporting events” were the main reasons. Furthermore, athletes from various sports reported that a reduction in negative menstrual-related symptoms is a positive side-effect of using HC (Martin et al., 2018; Clarke et al., 2021). However, negative side-effects such as weight gain, irregular periods, and mood swings are also reported with HC use and are mentioned as reasons for disuse of HCs (Martin et al., 2018; Clarke et al., 2021). Although the above-mentioned studies provide initial insights into the self-reported reasons and side-effects experienced by athletes using HCs, information about the self-perceived influence of HC use on endurance training among athletes of different age groups and performance levels, and whether their experiences differ from non-HC users, is lacking.

In the previous study by Solli et al. (2020), 17% of HC using athletes reported HCs to have a positive effect on their physical fitness or performance, while only 5% experienced negative effects. Since this study provided limited insights into differences between types of HC preparations, reasons for HC use, and how the HC affected training and performance, the present study aimed to build upon the work of Solli et al. (2020), by investigating HC users in more detail. Therefore, the aims of the current study were to: (1) investigate the prevalence of different types of HC (progestin-only vs. combined HCs) used by female cross-country (XC) skiers and biathletes competing at a national and/or international level; (2) explore the athletes' reasons for HC use; (3) compare negative symptoms related to the HC-/menstrual cycle experienced by HC users and non-HC users; and (4) characterize the self-perceived influence of HC use on training and performance.

## Materials and Methods

The current study is part of The Female Endurance Athlete (FENDURA) project, which is led by the School of Sport Sciences at UiT The Arctic University of Norway, in collaboration with the Norwegian University of Science and Technology, the Norwegian Olympic Committee (Olympiatoppen), the Norwegian Ski Federation, and the Norwegian Biathlon Federation. The overall aim of the FENDURA project is to increase the knowledge on how female-specific aspects, such as the menstrual cycle and HC use, influence training and performance among female endurance athletes.

### Participants

From May 2020 to September 2020, 221 Norwegian competitive female XC skiers and biathletes were invited to complete a questionnaire about their menstrual cycle and HC use. The recruitment process was performed in collaboration with coaches and staff members of the Norwegian Ski Federation and the Norwegian Biathlon Federation, and by approaching athletes directly. The inclusion criteria were as follows: (1) competing at a national or international level; (2) above 18 years of age; (3) training systematically in their sport for at least 3 years. Of the athletes invited, 115 athletes completed the questionnaire. Of these, one athlete was excluded from the analysis due to missing consent form and one athlete was excluded due to low age (<18 years). Thus, 113 responses were included in the analysis; of these, 50 athletes were biathletes and 63 were XC skiers. Overall, the total sample included 51 juniors (born ≥2000) with an average age of 19 (range from 18 to 20) years and 62 seniors (born ≤ 1999) with an average age of 24 (range from 20 to 32) years. Based on the framework by McKay et al. (2022), 30 athletes (10 juniors and 20 seniors) belonging to a national team (recruit, junior, elite) were classified as Tier 4 and 5, while 83 athletes were classified as national level athletes (Tier 3) based on their training volume being within 20% of the international athletes (Table 1). The study was evaluated by the Regional Committees for Medical and Health Research Ethics (REK) and approved by the Norwegian Social Science Data Services (NSD). All participants were given oral and written information about the study, before providing their written informed consent to participate.

**Table 1:** Participant characteristics stratified by type of hormonal contraceptive (HC), and by use and non-use of HC (mean and SD).

**Characteristics information**	**Progestin-only HC users** **(*n* = 49)**	**Combined HC users** **(*n* = 28)**	**All HC users** **(*n* = 77)**	**Non-HC users** **(*n* = 36)**	**Seniors** **(*n* = 62)**	**Juniors** **(*n* = 51)**	**National team athletes** **(*n* = 30)**	**Non-national team athletes** **(*n* = 83)**
Age (y)	21.3 (2.7)	22.2 (4.0)	21.6 (3.2)	20.8 (3.1)	23.5 (2.9)**	18.8 (0.7)	23.2 (3.9)**	20.7 (2.6)
Body height (cm)	168.6 (5.0)	168.1 (4.3)	168.4 (4.7)	169.3 (6.4)	168.4 (5.3)	169.0 (5.4)	169.8 (4.9)	168.3 (5.4)
Body mass (kg)	61.5 (5.3)	60.3 (3.3)	61.1 (4.7)**	63.5 (4.7)	61.2 (4.6)	62.6 (4.9)	63.2 (5.0)	61.4 (4.7)
Annual training volume (hr/year)	611.5 (113.1)*	667.8 (124.8)	632.2 (119.8)	644.7 (122.4)	679.3 (122.5)**	582.8 (93.9)	725.9 (120.6)*	603.4 (102.7)

Significant differences between groups (*P < 0.05 and **P < 0.01).

### Questionnaire

**Figure 1:**
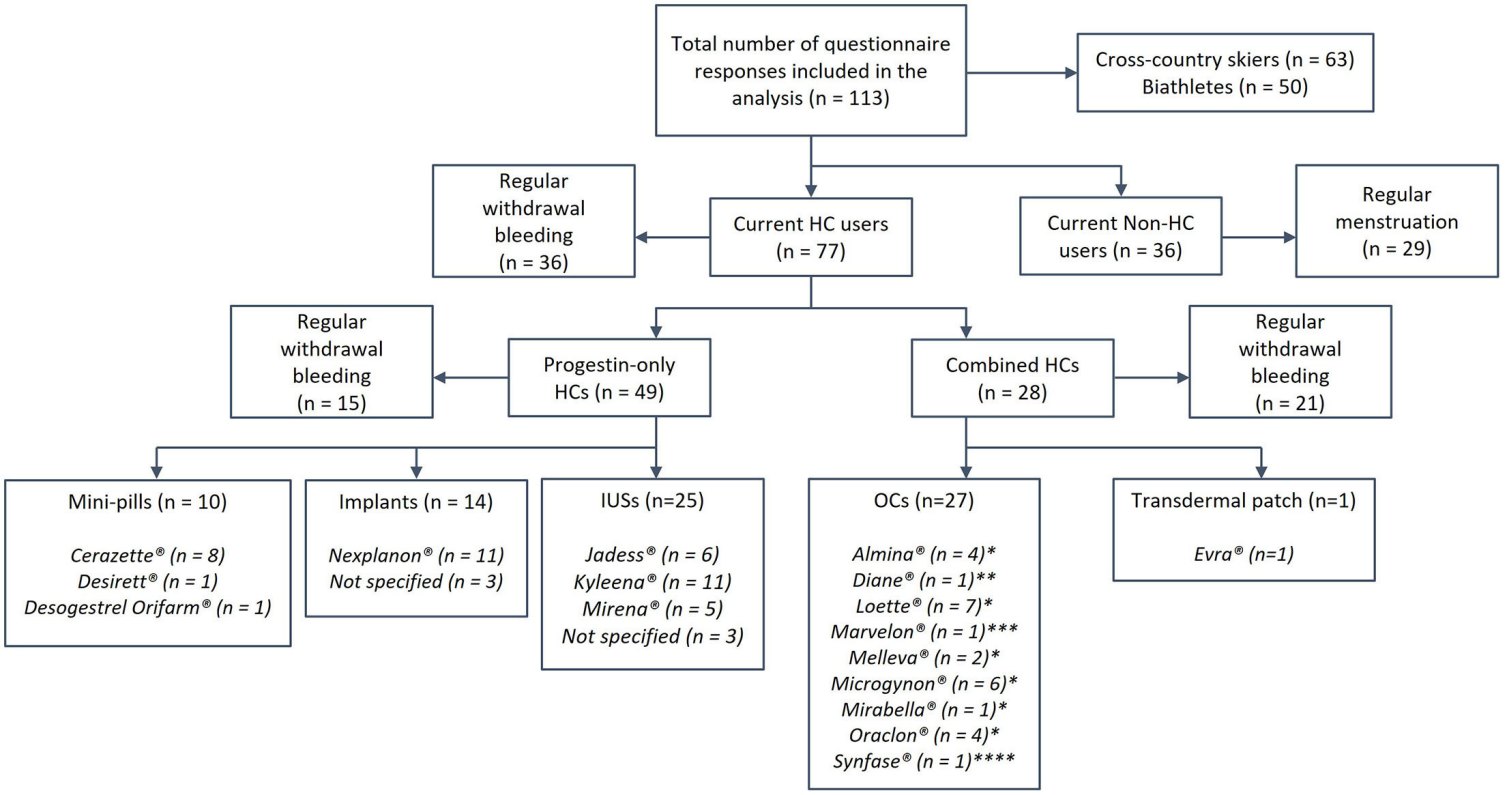
The prevalence of type, delivery methods, and preparations of hormonal contraceptives (HCs) used. Concentration of synthetic hormones in the different delivery methods: Mini-pills, 75 μg Desogestrel; Implants, ~40 μg Etonogestrel in 24 h over 3 y; Intrauterine devices (IUSs), 6–15 μg Levonorgestrel in 24 h over 3–6 y; Oral contraceptives (OCs), * 20–30 μg Ethinyl estradiol (EE) and 100–150 μg Levonorgestrel, **35 μg EE and 2,000 μg Cyproterone acetate, ***30 μg EE and 150 μg Desogestrel, ****35 μg EE and 500–1,000 μg Noretisterone; Transdermal patch, 33.9 μg EE and 203 μg Norelgestromin in 24 h.

The questionnaire used in the current study was based on the previously published questionnaire by Solli et al. (2020) but modified based on consultations with an expert panel, including former athletes, physiologists, medical experts, a gynecologist, and coaches. The current questionnaire was validated in Norwegian for use among Norwegian athletes (Supplementary Table 1) and contained several questions, both closed- and open-ended, about HC use, reasons for use, and how HCs influence self-perceived training quality and performance. The questionnaire was designed to take ~15–30 min to complete. All participants reported age, body height, body mass, and total training volume over the preceding season (May 1st 2019, to April 30th 2020) (Table 1). Additionally, HC users reported information about which HC delivery method and brand they used (Figure 1). Athletes were grouped as either “current HC users” or “non-HC users,” and each group completed different sections of the questionnaire. Current HC users reported if they used HC for non-contraceptive reasons, and athletes who used HC for non-contraceptive reasons were also asked to state these reasons by answering an open-ended question. HC users were asked to report if they experienced any positive and/or negative influence of HC use on training and performance. All HC users who experienced a positive or negative influence on training/performance were then asked to specify these experiences through answering open-ended questions. Both HC users and non-HC users were asked if they had regular withdrawal bleedings/menstruation. Athletes with regular bleedings were asked if they had any negative menstrual-related symptoms (i.e., experienced negative symptoms in relation to their HC-/menstrual cycle), and to specify these symptoms by answering an open-ended question.

### Data Analysis

Responses to the open-ended questions were analyzed by the content comparative methods of analysis (Postholm, 2019). By employing an abductive analysis approach, these questions were coded and categorized by two authors of the current study (TE and GS), which were further discussed until a consensus was reached. Frequency analyses from the open-ended questions on symptoms and reasons for HC use were completed by counting codes in the different categories. A selection of responses, representing each category from the open-ended questions about “reasons for HC use” and “self-perceived influence of HC use on training/performance,” are provided as examples of the interpretation of the responses to the various open-ended questions (**Tables 3**, **5**). Quantitative data are presented as mean (SD), frequencies, or prevalence, and the statistical significance level was set at *P* < 0.05. The athletes were categorized based on HC use vs. non-HC use, as well as the type of HC (progestin-only vs. combined). We also stratified athletes based on age (senior vs. junior) and national team athletes (Tier 4 and 5) vs. non-national team athletes (Tier 3). Categorical and numerical data were analyzed using the Statistical Package for the Social Sciences (SPSS 26, IMB Corp, Armonk, NY, USA). Data were examined for normality distribution before analysis using a Shapiro–Wilk test and visual inspection of Q–Q plots and histograms. Independent sample *t*-tests and Mann-Whitney *U*-tests were used to examine between-group differences, with the latter test being used for data that were considered as non-normally distributed. The relationship between categorical variables was examined with Pearson's chi-square analysis, with Fisher's exact tests being used when any expected cell counts were <5, i.e., using a conservative approach (Field, 2013).

## Results

Detailed information about the athletes' characteristics are presented in Table 1.

### HC Use

Of all participants, 68.1% were currently using HC, of whom 63.6% used a progestin-only HC (IUS: 51%; implants: 28.6%; mini-pills: 20.4%), while 36.4% used combined HC (combined-OC: 96.4%; one patch-user). A lower proportion of progestin-only vs. combined users (30.6 vs. 75.0%, *P* < 0.001) reported having a regular withdrawal bleeding in connection with their HC use. Furthermore, 19.5% of the HC users had previously stopped using another HC due to experiencing a negative influence on physical fitness and/or performance. No difference in the prevalence of HC use or the type of HC were found between junior and senior athletes (HC use: *P* = 0.054; Type of HC: *P* = 0.596) or between national and non-national team athletes (HC use: *P* = 0.799; Type of HC: *P* = 0.074, for details see Supplementary Table 2).

### Reasons for HC Use

Detailed information about reasons for HC use is presented in Table 2 and a selection of statements is presented in Table 3. For detailed information about the juniors vs. seniors and national- vs. non-national team athletes see Supplementary Table 3). Among the 77 HC users, 59.7% reported that they used HC for non-contraceptive reasons, including 51% of the progestin-only HC users vs. 75% of the combined HC users (*P* = 0.039). For these athletes, menstrual symptoms were the most common reason (60% of the progestin-only HC users vs. 67% of the combined HC users, *P* = 0.762), while practical reasons were reported by 40% of the progestin-only HC users vs. 52% of the combined HC users (*P* = 0.553). Furthermore, 16% of the progestin-only HC users vs. 43% of the combined HC users (*P* = 0.056) reported compound reasons (i.e., symptoms influencing performance and/or for avoiding symptoms during competitions). Health-related reasons were reported by 8% of the progestin-only HC users vs. 19% of the combined HC users. When stratified based on age and performance level, non-contraceptive reasons for HC use were reported by 76.7% of the juniors vs. 48.9% of the seniors (*P* = 0.016), and by 61.9% of the national and by 58.9% of the non-national team athletes (*P* = 0.813). No differences were detected between junior vs. senior and between national vs. non-national team athletes when comparing the different non-contraceptive reasons for using HCs.

**Table 2:** Frequency and prevalence for hormonal contraceptive (HC) use.

**Reported reasons for HC use**	**Progestin-only HC users (*****n*** **= 25)**		**Combined HC users (*****n*** **= 21)**		**All HC users (*****n*** **= 46)**	
	**Frequency (*n*)**	**Prevalence, %**	**Frequency (*n*)**	**Prevalence, %**	**Frequency (*n*)**	**Prevalence, %**
**Menstrual symptoms**	15	60	14	67	29	63
Unspecified pain	11	44	13	62	24	52
Heavy bleeding	7	28	2	10	9	20
Reduced physical fitness/emotional feelings	3	12	0	0	3	7
**Practical**	10	40	11	52	21	46
Avoid menstruation during competitions	6	24	9	43	15	33
Cessation of menstruation	4	16	0	0	4	9
Control of the cycle	2	8	3	14	5	11
Regular menstrual cycle	0	0	1	5	1	2
**Compound reasons**	4	16	9	43	13	28
Symptoms influence performance	4	16	4	19	8	17
Avoid symptoms during competitions/training camps	2	8	5	24	7	15
Health-related reasons^*^	2	8	4	19	6	13

* *No detailed information due to low n*.

*No significant differences progestin-only HC users and combined HC users when comparing reasons (symptoms: P = 0.762, Cramer's V = 0.069; practical: P = 0.553, Cramer's V = 0.124; compound reasons: P = 0.056, Cramer's V = 0.297)*.

*No significant differences between juniors and seniors when comparing reasons (symptoms: P = 0.760, Cramer's V = 0.045; practical: P = 0.139, Cramer's V = 0.218; compound reasons: P = 0.743, Cramer's V = 0.048)*.

*No significant differences between national and non-national team athletes when comparing reasons (symptoms: P = 0.739, Cramer's V = 0.080; practical: P = 0.203, Cramer's V = 0.188; compound reasons: P = 0.469, Cramer's V = 0.142)*.

**Table 3:** Selection of responses: reasons for hormonal contraceptive (HC) use.

**Participants' reasons for using HC**			
**Symptoms**	**Practical**	**Compound reasons**	**Health-related**
“I use it to reduce severe menstrual cramps” “I suffered from prolonged, heavy, frequent bleeding that disturbed my everyday life. It became easier to handle with combined oral contraceptives. My menses became lighter, and my bleedings shorter with longer duration between each bleeding” “To reduce the amount of bleeding, pain and a consistent bad feeling” “Heavy bleedings, and some menstrual cramps” “Severe menstrual cramps” “Bloating, ailments, and abdominal pain” “To reduce menstrual pain”	“To get continuity in my menstruation, the ability to skip menstruation and as contraception” “To avoid menstruation at competitions/training camps etc.” “Have control/avoid menstruation during training and competition” “Loss of menstruation” “Can postpone my period when I am competing or at training camps” “The possibility to skip menstruation, but the main reason is contraception” “To prevent menstruation and therefore make it more convenient, such that I do not need to think about it”	“To regulate when I have menstruation because I get painful cramps that I feel reduce my performance. Because of this, I can avoid having my period during important competitions” “To avoid bleeding at training camps and during training as it is both annoying and painful. The intrauterine system reduces my menstrual pains” “… Also had very strong menstrual cramps, and needed to be able to control my menstruation in relation to competitions etc…” “To avoid menstrual cramps during important competitions” “Severe and unpredictable menstrual cramps especially the first four days, which reduce my performance extremely” “[I] had so much menstrual pain before I started on combined oral contraceptives that I could not train” “Abdominal pain. I have struggled a lot with stomach cramps due to menstruation, which sometimes affected my performance”	“Both for contraception and due to low production of estrogen” “… polycystic ovary syndrome. I had a too low estrogen level” “Iron deficiency”

### Symptoms Related to HC- and Menstrual Cycle

A detailed overview of the athletes' self-reported menstrual-related symptoms is presented in Table 4. Of the HC users with regular withdrawal bleeding, 80 and 85.7% of the respective progestin-only and combined HC users reported having menstrual-related symptoms. By comparison, 86.2% of the non-HC users who reported regular menstruation experienced menstrual symptoms. There were no significant differences between progestin-only and combined HC users or between all HC users and non-HC users (Supplementary Table 4).

**Table 4:** Prevalence of reported menstrual symptoms for progestin-only and combined hormonal contraceptive (HC) users, and for all HC users and non-HC users.

**Symptoms**	**Progestin-only HC users (*****n*** **= 12)**		**Combined HC users (*****n*** **= 18)**		**All HC users (*****n*** **= 30)**		**Non-HC users (*****n*** **= 25)**	
	**Frequency** **(*n*)**	**Prevalence,** **%**	**Frequency** **(*n*)**	**Prevalence,** **%**	**Frequency** **(*n*)**	**Prevalence,** **%**	**Frequency** **(*n*)**	**Prevalence,** **%**
**Physical**								
Stomach cramps/abdominal pain	6	50	16	89	22	73	13	52
Back pain	4	33	5	28	9	30	11	44
Heavy bleeding	4	33	4	22	8	27	4	16
Tiredness/fatigue/lethargy	2	17	1	6	3	10	4	16
Nausea/sickness/vomiting	0	0	2	11	2	7	4	16
Unspecified pain/cramps	2	17	0	0	2	7	3	12
Bloating/Other stomach problems	2	17	0	0	2	7	3	12
Reduced physical fitness	0	0	0	0	0	0	2	8
Muscle- and/or joint ache	2	17	2	11	4	13	2	8
Sweating/hot flushes	0	0	1	6	1	3	1	4
Hunger/increased appetite	1	8	0	0	1	3	1	4
Sore breasts	1	8	0	0	1	3	1	4
Headache	1	8	1	6	2	7	0	0
**Emotional**								
Mood changes/swings	4	33	3	17	7	23	9	36
Demotivated/sad/depressed	1	8	1	6	2	7	2	8
Flustered/Unfocused	0	0	0	0	0	0	2	8

### HC Use and Self-Perceived Influence on Training and Performance

Detailed information about HC use and self-perceived influence on training and performance is presented in Supplementary Table 3. Of all HC users, 49.4% experienced a solely positive influence on training and/or performance, including 51% of the progestin-only HC users and 46.4% of the combined HC users. In total, 31.2% of the HC users experienced neither a positive nor a negative influence (i.e., “neutral”) on training and/or performance, while 14.3 and 5.2% experienced a mixed and a solely negative influence, respectively. There were no significant differences between progestin-only and combined HC users when investigating the self-perceived influence on training and performance (*P* = 0.942). No differences were detected between junior vs. senior (*P* = 0.788) and national vs. non-national team athletes (*P* = 0.379) when investigating the athletes' perception of how HCs influenced training and performance.

## Discussion

The current study explored the prevalence and self-perceived experiences with the use of hormonal contraceptives in Norwegian competitive endurance athletes. Our main findings were as follows: (1) 68% of the examined athletes used HC, with 64% of these using a progestin-only type; (2) 60% of the HC users reported non-contraceptive reasons for HC use, with menstrual symptoms as the main reason (i.e., 60% of the progestin-only HC users and 67% of the combined users; non-significant difference); (3) of the HC users with regular withdrawal bleeding, 80% of the progestin-only and 86% of the combined HC users experienced negative menstrual-related symptoms (non-significant difference), which was similar to the non-HC users (non-significant); (4) there were no significant differences in how the use of HC was perceived to influence training and/or performance between progestin-only and combined HC users, and the majority (81%) of all HC users experienced a solely positive or no influence, of using HC on training and/or performance.

### HC Use

The 68% prevalence of HC use in this study is higher than the ~50% prevalence reported in earlier studies on athletes from various sports (Martin et al., 2018; Oxfeldt et al., 2020; Solli et al., 2020; Clarke et al., 2021; Nolan et al., 2022). Furthermore, over 60% of HC users in the current study employed a progestin-only type of HC, which differs from previous studies where most (61–74%) athletes used a combined type (Martin et al., 2018; Oxfeldt et al., 2020; Solli et al., 2020). This prevalence of progestin-only HC use (64%) was substantially higher than the 38% reported in Solli et al. (2020), who investigated the same population of Norwegian XC skiers and biathletes. The reason for this difference could be the general increase in long-acting contraceptives, including implants and IUS, found in the general Norwegian population (Furu et al., 2021). Based on the Norwegian Prescription Database, the investigation by Furu et al. (2021) showed an increase in the use of implants and IUS from 8 to 26% from 2015 to 2020 in women aged 20–24 years. The reason for this increase may be that public health nurses and midwives were authorized to prescribe all hormonal contraceptives for women 16 years and older from 2016, including implants and IUS. Furthermore, the reason for the higher prevalence of progestin-only HC could be the ease of use, which is reported as the most common reason for athletes' choice of type/delivery method in the study by Martin et al. (2018). Accordingly, long-acting HCs as implants and IUS, have been preferred because of their high efficacy and ease of use (Burke, 2011). However, there is limited research examining how different types of HC, especially progestin-only types, affect athletic performance (Martin and Elliott-Sale, 2016; Clarke et al., 2021). The rapid increase in the use of progestin-only HCs, particularly long-acting HCs, in endurance athletes is interesting since a significantly higher incidence of negative side-effects has been reported in progestin-only compared to combined HCs (Martin et al., 2018). However, the positive effects of long-acting HCs on athletes' training quality and performance may potentially outweigh any negative side-effects. Future studies should seek to answer this question, in addition to directing more attention to the effects of long-acting HCs on training and performance.

### Reasons for HC Use

Sixty percent of the HC users reported that they used HCs for non-contraceptive reasons. Interestingly, this included a significantly higher proportion of combined HC users (75%) compared to progestin-only HC users (51%). The reason for the disagreement is unclear since no differences were detected in the reported reasons for HC use between the two groups. Reduction in negative menstrual-related symptoms was the main reason for HC use in both groups, with athletes stating (Table 3) to use HC “*To reduce the amount of bleeding, pain and a consistent bad feeling*,” and to reduce “*Bloating, ailments, and abdominal pain*.” Such reduction of menstrual symptoms, as well as “practical reasons” such as the ability to predict and/or change the HC-/menstrual cycle, and cessation of menstruation are all positive effects of HC use reported in previous studies (Martin et al., 2018; Oxfeldt et al., 2020; Clarke et al., 2021). However, these latter studies have mainly reported practical reasons as the most common non-contraceptive reason for HC use. Our findings of athletes reporting compound reasons (Table 3), highlight that some athletes use HC to reduce or avoid negative menstrual-related symptoms because they perceive this to interfere negatively with their training and performance.

### Symptoms Related to HC- and Menstrual Cycle

In the current study, only HC users with regular withdrawal bleeding were asked about having negative menstrual-related symptoms, which excluded nearly 70% of the progestin-only HC users. Our findings indicate that a large portion of the progestin-only HC users experiences irregular or cessation of bleeding, which is a known reported side-effect of using progestin-only HC (Burke, 2011). Of the athletes with regular withdrawal bleeding, negative menstrual-related symptoms similar to the experiences of non-HC users, occurred in both progestin-only and combined HC users (with no differences between groups), which is in line with previous studies (Solli et al., 2020; Clarke et al., 2021). However, Clarke et al. (2021) speculated that although HC users experience negative menstrual-related symptoms, they may experience decreased duration or severity of symptoms. In addition, Findlay et al. (2020) highlighted that some athletes in their study used HCs to manage their negative menstrual-related symptoms. In the current study, HC users were not asked if they had fewer/lighter menstrual symptoms compared to before they started using HC. However, responses such as “*[I] had so much menstrual pain before I started on combined oral contraceptives that I could not train*” (Table 3), “*[I have] slightly less pain than I had without contraception. This makes it easier to complete workouts…*” (Table 5) suggest that athletes' negative menstrual-related symptoms may have reduced in severity as a result of HC use.

**Table 5:** Selection of responses: how hormonal contraceptives (HCs) influence training and/or performance.

**Participants' experiences about how HCs influence training and/or performance**		
**Perceived a solely positive influence**	**Perceived a mixed influence**	**Perceived a solely negative influence**
**Influence on training:** “I do not have as much pain and heavy bleeding anymore. What's more, I have not been as exhausted or tired as before I started on combined OC, therefore I feel better when training” **Influence on performance:** “I have no pain and I'm not nauseous on competitions days anymore, feelings that I used to have before” **Influence on performance:** “Not directly on performance, but due to my previous menstrual problems, hormonal contraception has made it easier for me because I can avoid the negative menstrual symptoms” **Influence on training:** “I do not have as much menstrual pain with HC, which makes it easier to train and compete” **Influence on performance:** “HC causes less bleeding and less pain during menstruation, which has a positive effect related to my performance. It is easier to give maximum at competitions when I do not have severe pain. I do not have to take paracetamol before competitions” **Influence on training:** “To a positive degree, so that I could postpone menstruation until after a competition or reduce pain to perform better without too many distractions” **Influence on performance:** “I avoid pain, performs better. No need to think about changing sanitary pads/tampons or about bleeding through them” **Influence on training:** “[I have] slightly less pain than I had without contraception. This makes it easier to complete workouts. In addition, I bleed less. When I used OC, I had no pain or bleeding, but was often in a bad mood” **Influence on training:** “[I] struggled a lot with menstrual pain before, but after I started on the implant, this has decreased. This means that I am not knocked out when I have my period and can train as normal”	**Positive influence on training:** “I avoid menstruation, as well as menstrual pain” **Positive influence on performance:** “To avoid the menstrual cycle which can affect the hormone balance in the body. For example, it prevents me feeling very emotional for some periods, etc., which I can imagine could affect both training and performance” **Negative influence on performance:** “I do not get the natural answer that my body functions properly as it should during a normal menstrual cycle” **Positive influence on training:** “Higher iron levels, more stable body and fewer mood swings” **Negative influence on training:** “Easier to get nauseous” **Positive influence on training/performance:** “Predictable menstruation and the possibility to postpone it if it does not fit with competitions” **Negative influence on training/performance:** “Weight gain and irregular bleeding the first couple of months” **Negative influence on training:** “I can experience menstrual symptoms of pain and intermittent bleeding to varying degrees” **Positive influence on performance:** “In the period after menstruation, I feel stronger and fresher” **Positive influence on training:** “I got less bleeding and pain during menstruation when I first started on OC. In recent years no bleeding” **Negative influence on training:** “No bleeding in recent years has been practical in terms of training and competition performance. However, without (regular) menses, it is more difficult to confirm that I am not pregnant and that I have an adequate energy intake” **Positive influence on performance:** “No bleeding is practical”	**Influence on training:** “Somewhat more pain during menstruation” **Influence on training:** “I have negative experiences with mini-pills, but now when I use an implant, I have some intermittent bleeding and sometimes pain. When I get menstrual cramps, I think it is extra difficult to exercise and I am not always in shape to train” **Influence on performance:** “The days I have menstrual pain or heavy bleeding are much harder and makes it more difficult to perform optimally” **Influence on training:** “Irregular menstruation due to IUS. When I used OC, it was regular. What's more, stronger menstrual pain now than with using OC” **Influence on performance:** “When I used OC, I skipped menstruation a few times to avoid menstruation around important competitions. I cannot control this with IUS, so I think that is a negative point. At the same time, it is now very unpredictable when I will get my period” **Influence on training:** “Lethargic, and physically heavy. Harder to train. I lose the feeling of being ‘light’ in my body” **Influence on performance:** “Loses the feeling of being in shape, strong/fast and ‘light’ in my body”

### HC Use and Self-Perceived Influence on Training and Performance

No differences between progestin-only and combined HC users were detected when we examined the self-perceived influence of HC use on training and performance. Furthermore, nearly half of the athletes (51% of the progestin-only HC users and 46% of the combined users) experienced only a positive influence of HC use on training and/or performance. This is much higher than the 17% reported in Solli et al. (2020). While Solli et al. (2020) did not provide any further explanations for these positive effects, several of the athletes in our study stated that their perceived positive influence on training and/or performance was due to lighter, or absence, of negative menstrual-related symptoms (Table 5), e.g., “*I do not have as much menstrual pain with HC, which makes it easier to train and compete*” and “*[I] struggled a lot with menstrual pain before, but after I started on the implant, this has decreased. This means that I am not knocked out when I have my period and can train as normal*.” Based on the athletes' experiences in this study, it appears that a reduction of menstrual-related symptoms increases the perceived training quality, which might be an important positive effect from HC use in many athletes. Still, ~30% of the athletes did not experience any influence (i.e., “neutral”) of HC use on training and performance, while 14% experienced a mixed (i.e., both positive and negative) influence by using HC. In addition, 5% had solely negative experiences, which is in line with Solli et al. (2020). The negative influence reported by athletes in the current study were mostly related to perceived side-effects such as irregular bleeding or stronger negative menstrual-related symptoms.

Overall, the findings in this study emphasize that athletes use HC to improve their perceived training quality due to complex negative menstrual-related symptoms. Furthermore, our findings indicate large individual variations in response to HC use, probably due to the athletes' unique hormonal profile and their reaction to the composition of synthetic hormones in different types and brands of HCs (Elliott-Sale et al., 2021). The individual response to HC use also highlights the importance of proper communication between coaches, athletes, and medical staff, as well as the need to monitor health and performance when starting on a new HC to detect potential changes (Findlay et al., 2020; Solli et al., 2020; Höök et al., 2021).

### Strengths and Limitations

A strength of the current study is the inclusion of national team athletes, which in XC skiing and biathlon includes athletes of world-class level. The high athletic level of these participants is valuable for generalization of the findings across different groups of elite endurance athletes. No differences were found between the national and non-national team athletes, suggesting that these results are representative of competitive endurance athletes of different levels. However, the sole focus on only biathletes and XC skiers might limit the generalizability of these findings to women competing in other endurance sports. Furthermore, the analysis of the open-ended questions provides complementary qualitative insight when analyzing reasons for HC use and the athletes' perception of how HCs may influence their training and performance.

There are also some limitations of this study. First, only current HC users were asked about previous HC use and the perceived influence of HCs on training and performance. This may have excluded important information about non-HC users' previous experiences of HC use and reasons for discontinuation. Second, HC users were not asked about perceived side-effects related to HC use, which reduces the possibility to understand the relationship between a specific type of HC and perceived side-effects. Third, the questionnaire was originally designed for two main groups (HC users and non-HC users) where only athletes with regular menstruation (and withdrawal bleedings) were asked about negative menstrual-related symptoms. Optimally, all athletes should have been asked to state if they have symptoms. In addition, associations between the duration of HC use and negative menstrual-related symptoms experienced could not be conducted with our dataset. However, since this would have been interesting information in the discussion on how HC use influence negative menstrual-related symptoms, we recommend future studies to investigate this. Forth, it is a possibility that the lack of significant differences found in this study could be caused by the low proportion of combined HC users or athletes at an international level (Tier 4–5). Thus, further studies should aim to include even more athletes in their sample. Similar to the data presented in the current study, most of the results from previous studies are limited by the descriptive and comparative analyses.

## Conclusions

This study provides new insight into the prevalence and reasons for using HCs among Norwegian female endurance athletes, with in-depth knowledge on athletes' perceptions of how different types of HC influence their training and performance. Overall, we found 68% HC use in XC skiers and biathletes, which is a higher proportion then reported previously in other athlete populations. The highest proportion (64%) of athletes in the current study used a progestin-only HC, which follows the increasing trend in the general Norwegian female population. The most common non-contraceptive reason for using HC was to reduce negative menstrual-related symptoms. This substantiates the fact that many athletes use HC to avoid menstrual-related negative symptoms interfering with their training and/or competitions. These perspectives, alongside the observation of the high proportion of progestin-only HC users, provide important information for the development of specific guidelines and the direction for future research in this area.

## Data Availability Statement

The datasets presented in this article are not readily available because the dataset generated for this study is not publicly available due to privacy concerns. Requests for assessing the dataset should be directed to the corresponding author. Requests to access the datasets should be directed to TE, tina.engseth@uit.no.

## Ethics Statement

The studies involving human participants were reviewed and approved by REK, Regionale komiteer for medisinsk og helsefaglig forskningsetikk (Project ID: 135555). The patients/participants provided their written informed consent to participate in this study.

## Author Contributions

TE, GS, BM, TT, DN, ØS, and BW designed the study whereas TE collected data. TE, EA, GS, and BW performed the data and statistical analyses, and interpreted the results. TE wrote the first draft of the manuscript. All authors jointly revised the manuscript and approved the final version.

## Funding

This study was funded by the Tromsø Research Foundation (Project-ID: 19_FENDURA_BW) and UiT The Arctic University of Norway. The publication charges for this article have been funded by a grant from the publication fund of UiT The Arctic University of Norway.

## Conflict of Interest

The authors declare that the research was conducted in the absence of any commercial or financial relationships that could be construed as a potential conflict of interest.

## Publisher's Note

All claims expressed in this article are solely those of the authors and do not necessarily represent those of their affiliated organizations, or those of the publisher, the editors and the reviewers. Any product that may be evaluated in this article, or claim that may be made by its manufacturer, is not guaranteed or endorsed by the publisher.
