# Supplementary material for: Prevalence and Self-Perceived Experiences With the Use of Hormonal Contraceptives Among Competitive Female Cross-Country Skiers and Biathletes in Norway: The FENDURA Project
Source: Front Sports Act Living. 2022 Apr 14;4:873222. doi: 10.3389/fspor.2022.873222 (PMC9047044; doi:10.3389/fspor.2022.873222)
Supplement: Supplementary file 1 [file Table_1.pdf]

Supplementary Material

Supplementary Table 1. The questions have been translated into English for readability but have not been validated in English.

Questions	Answer options
1. Are you using any form of hormonal contraceptive?	- Yes<br>- No
2. Which delivery method do you use?	- Combined OCs<br>- Mini pills (progestin-only OCs)<br>- Vaginal rings<br>- Injections<br>- Transdermal patches<br>- Implants<br>- Intrauterine systems (IUSs)<br>- Other
3. What is the name of the product/brand that you use?	- Open-ended
4. Do you use hormonal contraceptive for reasons other than contraception?	- Yes<br>- No
5. If yes, please specify which reasons	- Open-ended
6. Have you stopped using a hormonal contraceptive because it negatively influenced your physical fitness or performance?	- Yes<br>- No
7. If yes, please specify why	- Open-ended
8. Do you experience any positive influence associated with exercise by using hormonal contraceptive?	- Yes<br>- No
9. If yes, please specify how	- Open-ended
10. Do you experience any negative influence associated with exercise by using hormonal contraceptive?	- Yes<br>- No
11. If yes, please specify how	- Open-ended

12. Do you experience any positive influence associated with performance by using hormonal contraceptive?	- Yes<br>- No
13. If yes, please specify how	- Open-ended
14. Do you experience any negative influence associated with performance by using hormonal contraceptive?	- Yes<br>- No
15. If yes, please specify how	- Open-ended
16. Do you have regular bleedings?	- Yes<br>- No<br>- Yes, but use hormonal contraceptives<br>- No, but use hormonal contraceptives
17. Do you experience any negative symptoms related to your hormonal contraceptive cycle/menstrual cycle?	- Yes<br>- No<br>- I don't know
18. If yes, please specify which negative symptoms and when these symptoms occur in your cycle	- Open-ended

Supplementary Table 2. Prevalence of hormonal contraceptive use stratified by age and performance level.

	**Juniors (n=51)**	**Seniors (n=62)**	***P*-value and Cramer's *V***	**National team (n=30)**	**Non-national team (n=83)**	***P*-value and Cramer's *V***
Hormonal contraceptive	30 (58.8%)	47 (75.8%)	*P* = 0.054, Cramer's *V* = 0.181	21 (70.0%)	56 (67.5%)	*P* = 0.799, Cramer's *V* = 0.024
Progestin-only	18 (60.0%)	31 (66.0%)	*P* = 0.596, Cramer's *V* = 0.060	10 (47.6%)	39 (69.6%)	*P* = 0.074, Cramer's *V* = 0.204
Combined	12 (40.0%)	16 (34.0%)		11 (52.4%)	17 (30.4%)	

Supplementary Table 3. Reasons for hormonal contraceptive (HC) use and self-perceived influence of HC on training and/or performance stratified by age, performance level, and type of HC.

Reasons for HC use

HC users	Juniors (n=30)	Seniors (n=47)	*P*-value and Cramer's *V*	National team (n=21)	Non-national team (n=56)	*P*-value and Cramer's *V*	Progestin-only (n=49)	Combined (n=28)	*P*-value and Cramer's *V*	All HC users (n=77)
Use HC for non-contraceptive reasons	23 (76.7%)	23 (48.9%)	P = 0.016, Cramer's V = 0.276	13 (61.9%)	33 (58.9%)	P = 0.813, Cramer's V = 0.027	25 (51.0%)	21 (75.0%)	P = 0.039, Cramer's V = 0.235	46 (59.7%)
Symptoms	14 (60.9%)	15 (65.2%)	P = 0.760, Cramer's V = 0.045	9 (69.2%)	20 (60.6%)	P = 0.739, Cramer's V = 0.080	15 (60.0%)	14 (66.7%)	P = 0.762, Cramer's V = 0.069	29 (63.0%)
Practical	13 (56.5%)	8 (34.9%)	P = 0.139, Cramer's V = 0.218	4 (30.8%)	17 (51.5%)	P = 0.203, Cramer's V = 0.188	10 (40.0%)	11 (52.4%)	P = 0.553, Cramer's V = 0.124	21 (46.0%)
Compound reasons	7 (30.4%)	6 (26.1%)	P = 0.743, Cramer's V = 0.048	5 (38.5%)	8 (24.2%)	P = 0.469, Cramer's V = 0.142	4 (16.0%)	9 (42.9%)	P = 0.056, Cramer's V = 0.297	13 (28.0%)
Health	3 (13.0%)	3 (13.0%)	Low n	1 (7.7%)	5 (15.2%)	Low n	2 (8.0%)	4 (19.0%)	Low n	6 (13.0%)

Self-perceived influence on training and/or performance

HC users	Juniors (n=30)	Seniors (n=47)	*P*-value and Cramer's *V*	National team (n=21)	Non-national team (n=56)	*P*-value and Cramer's *V*	Progestin-only (n=49)	Combined (n=28)	*P*-value and Cramer's *V*	All HC users (n=77)
Solely positive	16 (53.3%)	22 (46.8%)	P = 0.788, Cramer's V = 0.124	12 (57.1%)	26 (46.4%)	P = 0.379, Cramer's V = 0.189	25 (51.0%)	13 (46.4%)	P = 0.942, Cramer's V = 0.088	38 (49.4%)
Neutral	10 (33.3%)	14 (29.8%)		4 (19.0%)	20 (35.7%)		14 (28.6%)	10 (35.7%)		24 (31.2%)
Mixed	3 (10.0%)	8 (17.0%)		3 (14.3%)	8 (14.3%)		7 (14.3%)	4 (14.3%)		11 (14.3%)
Solely negative	1 (3.3%)	3 (6.4%)		2 (9.5%)	2 (3.6%)		3 (6.1%)	1 (3.6%)		4 (5.2%)

Supplementary Table 4. Regular withdrawal bleeding/menstruation and symptoms, stratified by age, performance level, type of HC, as well as all HC users and non-HC users.

**HC users**	**Juniors (n=30)**	**Seniors (n=47)**	***P*-value and Cramer's *V***	**National team (n=21)**	**Non-national team (n=56)**	***P*-value and Cramer's *V***	**Progestin-only (n=49)**	**Combined (n=28)**	***P*-value and Cramer's *V***	**All athletes**	**All HC users (n=77)**	**Non-HC users (n=36)**	***P*-value and Cramer's *V***
Regular withdrawal bleeding	14 (46.7%)	22 (46.8%)	P = 0.990, Cramer's V = 0.001	12 (57.1%)	24 (42.9%)	P = 0.263, Cramer's V = 0.128	15 (30.6%)	21 (75.0%)	P < 0.001, Cramer's V = 0.428	Regular withdrawal bleeding/ menstruation	36 (46.8%)	29 (80.6%)	P < 0.001, Cramer's V = 0.319
							**Progestin-only (n=15)**	**Combined (n=21)**			**All HC users (n=36)**	**Non-HC users (n=29)**	
Report symptoms							12 (80.0%)	18 (85.7%)	P = 0.785, Cramer's V = 0.200	Report symptoms	30 (83.3%)	25 (86.2%)	P = 1.000, Cramer's V = 0.112
